# Regulation of Endoplasmic Reticulum Stress Suppresses Early Pro‐Tumorigenic Events During *N‐Nitrosodiethylamine*‐Induced Hepatocarcinogenesis

**DOI:** 10.1002/jbt.71000

**Published:** 2026-06-28

**Authors:** Maya P. Shetty, Smita Hegde, Sanjay Bharati

**Affiliations:** ^1^ Department of Nuclear Medicine Manipal College of Health Professions, Manipal Academy of Higher Education Manipal India

**Keywords:** 8‐OHdG, DNA repair, endoplasmic reticulum stress, hepatocellular carcinoma, p53, UPR

## Abstract

Carcinogenesis is a dynamic, multistep process in which the initiation stage represents a critical and irreversible event that determines cellular fate. Although genetic alterations play a central role in tumor development, dysregulation of intracellular organelles such as the endoplasmic reticulum (ER) has emerged as an important contributor to cancer initiation. In the present study, we aimed to evaluate how modulation of ER stress affects the initiation phase of hepatocarcinogenesis. The initiation‐stage hepatocarcinogenic model was developed by administering a single dose of *N‐nitrosodiethylamine* (NDEA) (50 mg/kg b.w, i.p.) to male Wistar rats. To modulate ER activity, the endogenous chaperone inducer 1‐(3,4‐dihydroxyphenyl)‐2‐thiocyanatoethanone (BIX) was administered intraperitoneally (0.1 mg/kg body weight) for 2 weeks prior to NDEA treatment. The effect of ER modulation on pro‐tumorigenic events was assessed in terms of oxidative DNA damage, expression of inflammatory cytokines, p53, and cellular proliferation. Modulation of UPR by BIX was evaluated based on expression levels of *PERK, ATF6, CHOP*, and p‐PERK/PERK ratio. BIX treatment demonstrated marked attenuation of pro‐tumorigenic events as evidenced by significantly decreased levels of 8‐OHdG, TNF‐α, IL‐6, PCNA, and p53, which were upregulated in the NDEA‐treated group. Histopathological analysis further confirmed the preservation of hepatic architecture in the BIX‐treated group as compared with the NDEA group. In addition, significantly decreased expression of UPR markers in the BIX‐treated group, as compared to the NDEA group, confirmed inhibition of UPR activation. Collectively, these findings demonstrated that BIX‐mediated ER‐stress modulation effectively inhibited the initiation of NDEA‐induced hepatocarcinogenesis.

## Graphical abstract

Schematic illustration of the effect of BIX in attenuating the initiation stage of hepatocarcinogenesis. BIX pre‐treatment mitigated *N‐nitrosodiethylamine‐*induced ER stress, DNA damage, and downstream pro‐tumorigenic events during the initiation phase. It preserved DNA repair capacity, reduced inflammation and cellular proliferation, thereby inhibiting early carcinogenic initiation.

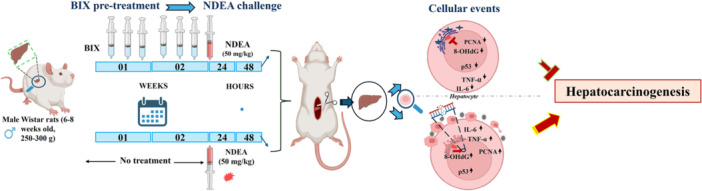

## 1 Introduction

Carcinogenesis is a dynamic process involving the sequential phases of initiation, promotion, and progression, with initiation representing a critical and often irreversible stage that determines cellular fate [1]. During this stage, normal cells encounter carcinogenic stimuli such as chemical agents, oxidative stress, metabolic disturbances, or inflammatory signals, resulting in DNA damage that can give rise to permanent genetic mutations if not accurately repaired [2]. These early mutations, together with epigenetic alterations, establish a pool of initiated cells that harbor heritable molecular defects while frequently maintaining a morphologically normal appearance.

Although these genetic alterations are commonly detected in normal tissues, only a small fraction progresses to malignancy, indicating that mutation alone is insufficient to drive tumor formation; rather, additional events might underlie cancer initiation and progression [3]. Among the various etiological factors associated with cancer development, exposure to environmental carcinogens is recognized as a major driver of carcinogenic initiation. Nitrosamines constitute an important class of such compounds, and among them, N‐nitrosodiethylamine (NDEA) is one of the most extensively studied experimental hepatocarcinogens. Following metabolic activation by cytochrome P450 enzymes, NDEA generates reactive ethylating intermediates that induce DNA adduct formation and genotoxic injury in hepatocytes, thereby triggering early molecular alterations associated with the initiation stage of hepatocarcinogenesis [4]. Additionally, NDEA also disrupts intracellular homeostasis and activates multiple stress‐response pathways, suggesting that cellular adaptation mechanisms might influence the fate of initiated hepatocytes [5].

Several studies have suggested that intracellular organelles play an important role in shaping the cellular response to carcinogenic stress and, consequently, in determining whether initiated cells remain dormant, undergo apoptosis, or progress toward malignancy [6, 7, 8]. Among these organelles, the endoplasmic reticulum (ER) has emerged as a critical regulatory hub due to its central roles in protein folding, lipid biosynthesis, and calcium homeostasis [9]. During carcinogenic stress, the ER activates a coordinated signaling network known as the unfolded protein response (UPR), which enhances cellular adaptability and promotes survival of initiated cells [10]. This response is mediated through three primary branches: PERK, IRE1α, and ATF6. Activation of the PERK pathway selectively reduces global protein synthesis while promoting metabolic adaptation, autophagy, and NRF2‐driven antioxidant signaling, thereby supporting the survival and stress tolerance of transformed cells [11]. Further, activation of the IRE1α pathway engages its downstream effector TRAF2, which activates JNK and NF‐κB‐dependent signaling, reinforcing pro‐survival transcriptional and inflammatory programs that enhance cellular adaptability and growth [12]. In parallel, ER‐associated oxidative protein‐folding processes increase the production of reactive oxygen species (ROS), which function as signaling molecules to modulate redox‐sensitive pathways, including MAPK, STAT3, and PI3K‐AKT‐mTOR, thereby promoting transcriptional plasticity and proliferative signaling [13, 14, 15]. Further, ATF6 complements these mechanisms by maintaining proteome stability during elevated biosynthetic activity [16].

Therefore, modulating the ER activity during carcinogenesis could be a promising strategy to impede carcinogenesis. There are several ER stress modulators that are known to modulate ER stress, and they have demonstrated positive outcomes in various disease conditions [17, 18, 19]. Most of these agents primarily function by modulating downstream components of the UPR, enhancing protein folding efficiency, or altering stress signaling pathways to restore ER homeostasis. However, such interventions indirectly influence stress adaptation levels rather than directly enhancing endogenous ER chaperone expression at the transcriptional level. Kudo et al. identified a new molecule known as 1‐(3,4‐dihydroxyphenyl)‐2‐thiocyanatoethanone (BIX), which selectively induces *GRP78/BiP* gene *via* activation of the ATF6 signaling pathway, thereby representing a novel approach to modulate ER stress [20]. It has been reported that BIX pre‐treatment increases cellular tolerance to ER stress and reduces ER stress‐induced inflammation and cell death, thereby contributing to the restoration of ER homeostasis [20, 21]. This was well demonstrated in several disease conditions such as cerebral ischemia, neurodegenerative disorders, rheumatoid arthritis, and diabetes [21, 22, 23, 24]. Considering this, in the present study, we aimed to evaluate how BIX affects the initiation phase of hepatocarcinogenesis.

## 2 Materials and Methods

### 2.1 Chemicals and Reagents

*N‐nitrosodiethylamine* (NDEA), 1‐(3,4‐dihydroxyphenyl)‐2‐thiocyanatoethanone (BIX), thiobarbituric acid (TBA), and 2′,7′‐Dichlorodihydrofluorescein diacetate were procured from Sigma‐Aldrich (St. Louis, MO, USA). TRIzol reagent, chloroform, isopropanol, and molecular‐grade ethanol were procured from Invitrogen (Thermofisher Scientific, Waltham, MA, USA). Rabbit IgG secondary antibody (65–6120), PERK (PERK‐101AP), p‐PERK (MA5‐15033), IL‐6 (BS0782R), IL‐10 (PA5‐85660), TNF‐α (BS2081R), 8‐OHdG (BS1278R), p53 (MA5‐12557), PCNA (MA5‐11358), and Maxima SYBR Green/ROX qPCR Master Mix were purchased from Thermofisher Scientific (Waltham, MA, USA). Nuclease‐free ultrapure water and dimethyl sulfoxide were purchased from Himedia Laboratories (Mumbai, India). All other reagents employed in the present work were of analytical grade and sourced from Sisco Research Laboratories Pvt. Ltd (Mumbai, India).

### 2.2 Animal Care and Experimental Design

Healthy male Wistar rats (250–300 g; 8‐12 weeks) were procured from the institutional central animal facility after receiving approval from the Institutional Animal Ethics Committee *(IAEC/KMC/119/2022)*. In accordance with Committee for Control and Supervision of Experiments on Animals (CPSEA) guidelines, India, animals were housed under standard environmental conditions with a temperature of 25°C ± 1°C, humidity 65%–80%, and a 12:12 h light and dark cycle. Before experimentation, all animals were acclimatized for a period of 1 week. These animals (*n* = 24) were randomly segregated into 4 groups, that is, CONTROL, BIX, NDEA, NDEA + BIX. Animals in the CONTROL group received an equivalent volume of vehicle (10% DMSO, intraperitoneally) throughout the study period. BIX group received BIX (0.1 mg/kg b.w., dissolved in 10% DMSO, i.p., thrice a week), throughout the study period. Animals in the NDEA group were treated with a single intraperitoneal injection of NDEA (50 mg/kg b. w., intraperitoneally). Animals in the NDEA + BIX group were subjected to NDEA and BIX treatment as described for the NDEA and BIX groups, respectively. The BIX administration in the respective groups started 2 weeks prior to the NDEA administration. All animals were fed with a standard diet and water *ad libitum*. Physiological parameters, including body weight and diet intake, were monitored throughout the study period.

### 2.3 UPR Gene Expression Analysis by Reverse Transcription Quantitative Real‐Time Polymerase Chain Reaction (RT‐qPCR)

#### 2.3.1 RNA Isolation and Complementary DNA (cDNA) Synthesis

Total RNA was extracted from hepatic tissue using TRIzol reagent (Invitrogen Life Technologies, USA) following the phenol‐chloroform method described previously [25], RNA samples with an absorbance ratio (OD 260/280) between 1.9 and 2.2 were used for reverse transcription. For cDNA synthesis, 2 μg of total RNA was converted to cDNA using the iScript cDNA synthesis Kit (Bio‐Rad Laboratories, Inc., CA, USA) as per the manufacturer's instructions.

#### 2.3.2 Primer Design

To evaluate changes in endoplasmic reticulum stress‐associated genes, specific UPR target genes like *GRP78/BiP*, *PERK, ATF‐6* and *CHOP* were selected, and primer sequences were designed using nucleotide sequences identified using NCBI BLAST software. The primers used in these studies were designed to span the exon‐exon junction to avoid genomic DNA amplification and target specificity. *GAPDH* was used as the housekeeping gene for normalization and the primer sequences are listed in the table (Table 1).

**Table 1:** Primer sequences used for quantification of UPR‐related genes.

Gene name	Genebank Accession no.	Primers	Product length (bp)
*Grp‐78/HSPA5*	NM_013083.2R	F: TCGGACGCACTTGGAATGAC R: CCAAATACGCCTCGGCAGTT	189
*EiF2/PERK*	NM_031599.2R	F: CGAAGTGACCGTGGAAGAC R: ATCCCACGTCCAAATCCCAC	198
*ATF‐6*	NM_001107196.1R	F: CCAGGTGGTGTCAGAGAACC R: GCAGGGCTCACACTAGGTTT	156
*Ddit3/CHOP*	NM_001109986.1R	F: TACACCACCACACCTGAAAGC R: TCAAAGGCGAAAGGCAGAGA	74

*Note:* All primers were designed for the species‐*Rattus norvegicus* using NCBI‐Primer‐blast‐tool.

#### 2.3.3 RT‐qPCR Amplification and Gene Expression Analysis

Quantitative analysis of UPR gene expression was performed using RT‐qPCR as described previously by Xu et al. [26]. Briefly, 20 ng of cDNA template was amplified in 20 µL of reaction mixture containing gene‐specific primers and SYBR green‐ROX master mix (ThermoFisher Scientific, Paisley, UK) in QuantStudio 8 in real‐time PCR systems (Applied Biosystems, Foster City, CA, USA). PCR amplification was initiated with a denaturation step at 95°C for 30 s. The reaction then proceeded through 40 cycles, each comprising denaturation at 95°C for 15 s followed by annealing at 60°C for 30 s. The specificity of the amplified products was analyzed using the melt curve analysis. Relative gene expression levels were determined using 2^‐ΔΔCt^ method. All samples were normalized to the housekeeping gene *GAPDH*, as its expression exhibited minimal variation across all experimental groups under the present experimental conditions, including NDEA‐induced hepatic stress. Following normalization, the fold change in target gene expression was calculated relative to the control group.

### 2.4 Quantitative Analysis of Endoplasmic Reticulum Stress, Oxidative DNA Damage 8‐OHdG, Tumor Suppressor Protein p53, Inflammatory and Cell Proliferation Markers Using ELISA

The expression of ER‐stress (PERK & p‐PERK), oxidative DNA damage (8‐OHdG), tumor suppressor (p53), inflammatory cytokines (IL‐6, IL‐10 & TNF‐α), and cell proliferation protein (PCNA) was quantified using ELISA as described previously [27]. In brief, antigens extracted from hepatic tissues were coated onto ELISA plates (Himedia, India) and incubated at 4°C for 12 h. The antigen‐coated plates were subjected to sequential washing using phosphate‐buffered saline containing Tween‐20 (PBSTw) to remove unbound antigens. The plates were then treated with 1% BSA as a blocking agent to prevent non‐specific binding. The antigen‐coated plates were treated with wash buffer and blocked with a blocking agent for non‐specific binding. After PBST wash, wells were treated with 100 µL primary antibodies such as PERK, p‐PERK, 8‐OHdG, p53, IL‐6, IL‐10, TNF‐α and PCNA (diluted to 1:1000). After 2 h of incubation, the plates were rinsed with PBST and 100 µL of secondary antibody (goat anti‐rabbit IgG, 1:3000 dilution) was added. After 1.5 h of incubation, 150 µL of TMB substrate was added to all the wells and incubated for half an hour. The enzymatic reaction was terminated by adding 50 µL of sulfuric acid (2 M). Quantification of protein expression levels was performed spectrophotometrically by recording absorbance at 450 nm with a multimode microplate reader (BioTek, Synergy H1, Agilent Technologies, USA).

### 2.5 Assessment of Antioxidant Defense Status in Hepatic Tissues

To perform enzymatic assays, hepatic tissue homogenates (10% w/v) were prepared in 0.1 M KH_2_PO_4_ buffer [28]. The extent of lipid peroxidation (LPO) was quantified by spectrophotometric measurement of the amount of malondialdehyde‐thiobarbituric acid (MDA‐TBA) chromophore, expressed as nmol formed per mg protein per minute [29]. Reduced glutathione (GSH) levels were determined spectrophotometrically from the formation of 2‐nitro‐5‐mercaptobenzoic acid (TNB), expressed as nmol per mg protein [30]. Glutathione reductase (GR) was assessed spectrophotometrically by measuring the amount of NADPH oxidized during the reduction of oxidized glutathione (GSSG), expressed as nmol NADPH consumed per mg protein per minute [31]. Glutathione peroxidase (GPx) activity was quantified spectrophotometrically by monitoring NADPH oxidation during the reduction of H_2_O_2_ to H_2_O, expressed as nmol NADPH consumed per mg protein per minute [32]. Superoxide dismutase (SOD) activity was determined by measuring the amount of enzyme required to scavenge superoxide radicals generated during photo‐oxidation of hydroxylamine per mg protein [33]. Reactive oxygen species (ROS) were estimated using 2,7‐dichlorofluorescein diacetate (DCFH‐DA). The non‐fluorescent probe DCFH‐DA undergoes intracellular hydrolysis to DCFH, which, upon oxidation by ROS, gets converted to the fluorescent compound dichlorofluorescein (DCF). Fluorescence intensity was measured spectrofluorimetrically and expressed as arbitrary fluorescence units (AFU).

### 2.6 Protein Estimation

The total protein concentration in the sample was quantified by Lowry's method [34]. In brief, proteins react with alkaline copper tartrate to form a cupric‐protein complex, which subsequently reduces the Folin‐Ciocalteu reagent to produce a blue molybdate chromophore. Bovine serum albumin was used as the standard.

### 2.7 Assessment of Liver Injury Markers

Liver injury was evaluated by measuring serum levels of alkaline phosphatase (ALP), aspartate aminotransferase (AST), and alanine aminotransferase (ALT) in animal blood samples using the Tulip Diagnostics kit (Santacruz, India).

### 2.8 Histological Assessment of Hepatic Tissues

Microscopic examination was performed to evaluate the histopathological changes in hepatic tissue sections after hematoxylin and eosin (H&E) staining. Briefly, hepatic tissues were formalin‐fixed (10%), dehydrated through ascending grades of alcohol and embedded in paraffin wax. Thin sections (5 μm) were cut using a rotary microtome (Leica RM2255, Germany) and stained with H & E and examined for histoarchitectural changes under a conventional optical microscope (Labomed Lx 300, USA).

### 2.9 Statistical Analysis

All measurements were expressed as Mean ± S.D. Homogeneity of variance was assessed with Levene's test. Normality of data was assessed using the Shapiro‐Wilk test. One‐way ANOVA was used for intergroup comparison, followed by Post hoc Tukey's test. A *p*‐value ≤ *0.05* was considered statistically significant. Statistical analysis was performed using Jamovi Desktop version 2.6.44.

## 3 Results

### 3.1 Alleviation of ER Stress Attenuated NDEA‐Induced Hepatic Injury

**Figure 1:**
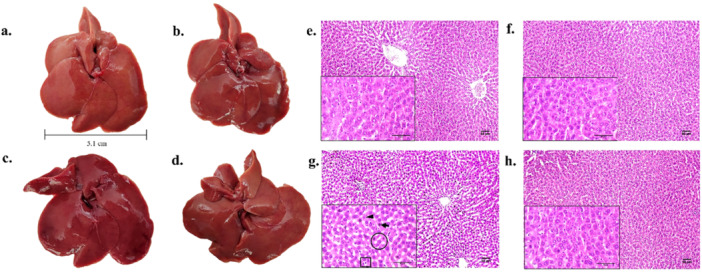
Effect of BIX on gross morphology and histopathology of hepatic tissues across different treatment groups. (a and b). Represent CONTROL and BIX groups showing normal hepatic morphology with intact lobular structure and distinct liver lobes. (c and d). NDEA and NDEA + BIX group showing normal hepatic structure. (e and f). Photomicrographs of CONTROL and BIX groups showing normal histoarchitecture with hepatocytes arranged in radial arrangements around the central vein and sinusoids. (g). Photomicrographs of NDEA group showing pyknotic nucleus (shrunken, densely condensed cell nuclei indicating irreversible cell death, represented by arrow; in inset), degenerated hepatocytes (represented by an arrowhead; in inset), cytoplasmic vacuolization (circle; in inset) and inflammatory cell infiltrates (represented by rectangle in inset). (h). Photomicrographs of NDEA + BIX group showing no visible signs of histopathological alterations, with preserved and intact hepatocyte organization. (Magnification: 100X with scale bar 50 μm; Inset image magnification: 400X with scale bar 50 μm).

NDEA administration resulted in significantly (*p* ≤ *0.05*) increased levels of liver injury markers (AST and ALT) as compared to the CONTROL group. In contrast, BIX pre‐treated animals on NDEA challenge exhibited significant protection against hepatic injury as evidenced by significantly (*p* ≤ *0.05*) decreased levels of liver injury markers as compared with NDEA group (Table 2). Gross morphological examination of hepatic tissues from NDEA and NDEA + BIX groups showed no alterations in comparison with CONTROL group (Figure 1a–d). Histopathological evaluation of liver sections in CONTROL and BIX groups demonstrated preserved hepatic architecture with radially arranged polyhedral hepatocytes surrounding central veins (Figure 1e,f). In contrast, NDEA group exhibited damaged hepatocytes, dilated sinusoids and inflammatory cell infiltrates as compared with CONTROL group (Figure 1g). Notably, NDEA + BIX group showed no observable alterations as compared with CONTROL group (Figure 1h).

**Table 2:** Effect of BIX on liver injury markers.

Parameter (U/L)	CONTROL	BIX	NDEA	NDEA + BIX
Aspartate aminotransferase (AST)	105.22 ± 3.9	105.1 ± 3.7	168.13 ± 6.7^a^, ^b^	121.3 ± 7.3^a^, ^b^, ^c^
Alanine aminotransferase (ALT)	40.20 ± 3.6	41.65 ± 0.5	113.7 ± 2.2^a^, ^b^	45.0 ± 1.6^c^
Alkaline phosphatase (ALP)	320.1 ± 2.1	326.3 ± 2.0	381.3 ± 5.3^a^, ^b^	340.3 ± 4.1^a^, ^b^, ^c^

*Note:* Data presented as mean ± S.D. Intergroup comparisons were performed using one‐way ANOVA followed by Tukey's HSD post hoc test. Statistical significance was considered at (*p* ≤ *0.05*) denoted by symbols;

^a^ w.r.t. CONTROL;

^b^ w.r.t. BIX group;

^c^ w.r.t. NDEA group.

### 3.2 BIX Mitigated NDEA‐Induced ER Stress and Activation of Unfolded Protein Response (UPR)

**Figure 2:**
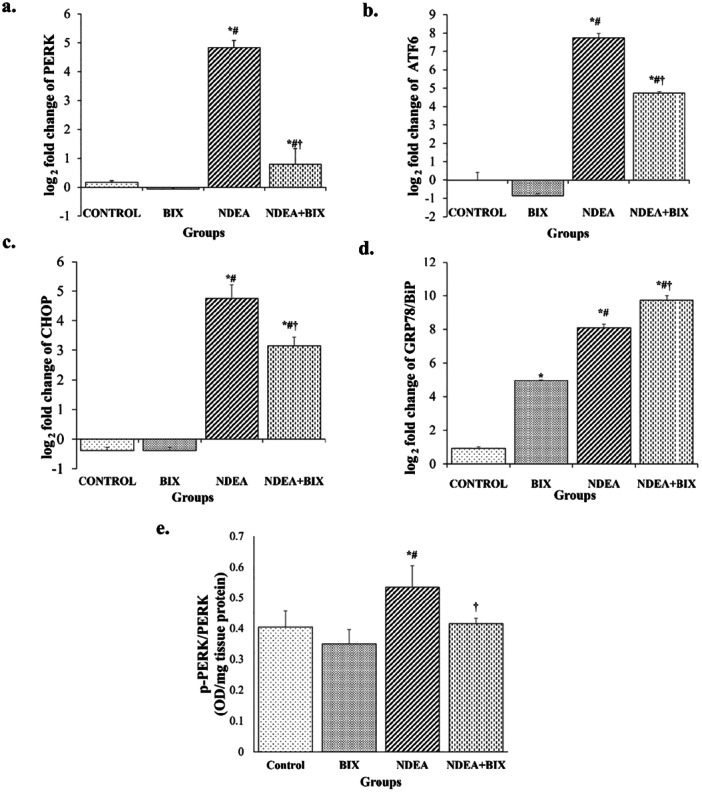
Effect of BIX on UPR genes during the initiation stage of hepatocarcinogenesis. (a). *PERK* gene expression. (b). *ATF6* gene expression. (c). *CHOP* gene expression. (d). *GRP78/BiP* gene expression. (e). p‐PERK/PERK ratio. (Data presented as mean ± S.D. Intergroup comparisons were performed using one‐way ANOVA followed by Tukey's HSD post hoc test. Statistical significance was considered at (*p* ≤ *0.05*) denoted by symbols: *w.r.t. CONTROL; ^#^w.r.t. BIX group; & ^†^w.r.t. NDEA group).

Quantitative RT‐PCR analysis of hepatic tissues after 48 h NDEA‐challenge showed significantly (*p* ≤ *0.05*) increased expression of ER stress‐associated genes like *PERK, ATF‐6* and *CHOP* as compared to CONTROL group. However, animals pre‐treated with BIX showed a significant (*p* ≤ *0.05*) decrease in the expression of *PERK, ATF‐6* and *CHOP* as compared with NDEA group (Figure 2a–c). Further, assessment of *GRP‐78/BiP* revealed a significant *(p* ≤ *0.05*) increase in its expression in the BIX alone‐treated group as compared to the CONTROL group, confirming the chaperone‐inducing potential of BIX. The expression of *GRP78/BiP* was significantly (*p* ≤ *0.05*) increased in the NDEA + BIX group as compared to the NDEA group (Figure 2d). These results were further supported by ELISA, where the p‐PERK/PERK ratio was significantly (*p* ≤ *0.05*) decreased in the NDEA + BIX group as compared to the NDEA group, highlighting the protective role of BIX in preventing ER stress and UPR.

### 3.3 BIX Treatment Suppressed NDEA‐Induced Oxidative Dna Damage, Hepatic Inflammation, Cellular Proliferation and p53 Expression

**Figure 3:**
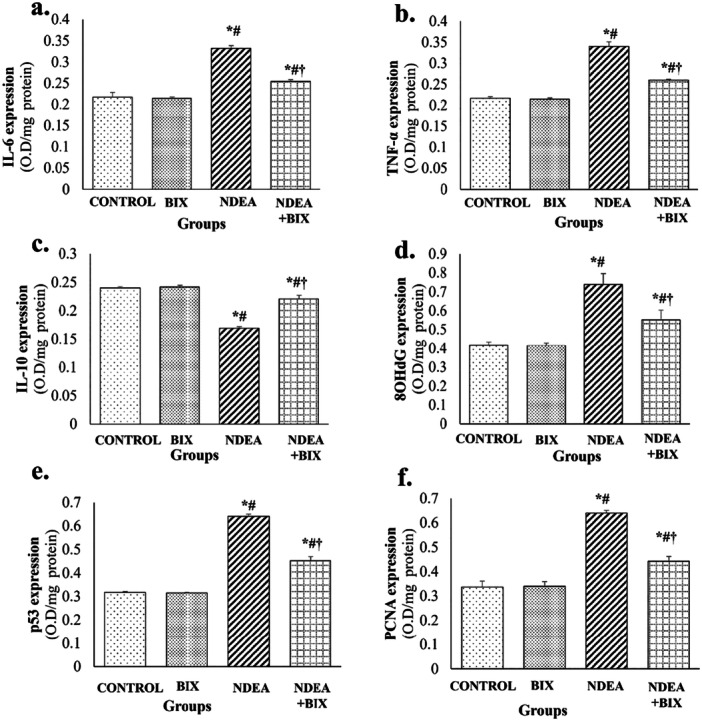
Effect of BIX on NDEA‐induced hepatic inflammation, oxidative DNA damage, proliferation, and p53 expression. (a and b). Quantitative analysis of pro‐inflammatory cytokines protein expression. (c). Quantitative analysis of anti‐inflammatory protein expression. (d). Quantitative analysis of 8‐OHdG protein expression. (e). Quantitative analysis of tumor suppressor protein (p53) expression. (f). Quantitative analysis of PCNA protein expression. (Data presented as mean ± S.D. Intergroup comparisons were performed using one‐way ANOVA followed by Tukey's HSD post hoc test. Statistical significance was considered at (*p* ≤ *0.05*) denoted by symbols: *w.r.t. CONTROL; ^#^w.r.t. BIX group; & ^†^w.r.t. NDEA group).

Quantitative analysis of hepatic tissues after 48 h NDEA‐challenge showed significantly (*p* ≤ *0.05*) increased expression of 8‐OHdG, p53, IL‐6, TNF‐α and PCNA as compared with CONTROL group. However, BIX‐pretreatment significantly (*p* ≤ *0.05*) decreased expression of IL‐6, TNF‐α, 8‐OHdG, p53, and PCNA as compared to NDEA group (Figure 3a,b,d–f). Further, it was observed that anti‐inflammatory cytokine IL‐10 was significantly (*p* ≤ *0.05*) increased in NDEA + BIX group as compared with the NDEA group (Figure 3c). No significant difference was observed in expression levels of 8‐OHdG, p53, IL‐6, TNF‐α and PCNA proteins in the BIX alone‐treated group as compared with the CONTROL group.

### 3.4 BIX Treatment Inhibited NDEA‐Induced Oxidative Stress and Lipid Peroxidation

**Figure 4:**
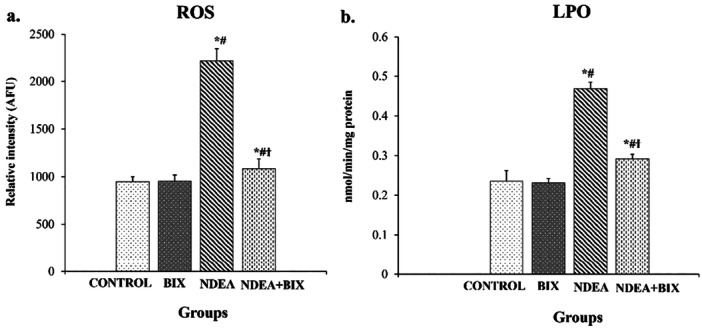
Effect of BIX on intracellular reactive oxygen species and lipid peroxidation levels. (a). Effect of BIX on levels of reactive oxygen species in different treatment groups. (b). Effect of BIX on levels of lipid peroxidation in different treatment groups. (Data presented as mean ± S.D. Intergroup comparisons were performed using one‐way ANOVA followed by Tukey's HSD post hoc test. Statistical significance was considered at (*p* ≤ *0.05*) denoted by symbols: *w.r.t. CONTROL; ^#^w.r.t. BIX group; & ^†^w.r.t. NDEA group).

NDEA‐challenged animals showed significantly (*p* ≤ *0.05)* increased levels of ROS and LPO as compared with the CONTROL group. In contrast, BIX pre‐treatment significantly (*p* ≤ *0.05*) decreased ROS and LPO levels as compared with NDEA group. No significant difference was observed in intracellular ROS and LPO levels in the BIX alone‐treated group as compared with the CONTROL group (Figure 4a,b).

### 3.5 BIX Treatment Restored the Hepatic Antioxidant Defense System

NDEA‐challenged animals showed significantly (*p* ≤ *0.05*) decreased levels of antioxidant enzymes like superoxide dismutase (SOD), glutathione peroxidase (GPx), and glutathione reductase (GR) as compared with CONTROL group. In addition, reduced glutathione (GSH) levels was also significantly (*p* ≤ *0.05*) decreased in CONTROL group following NDEA challenge. In contrast, BIX‐pre‐treated animals challenged with NDEA exhibited significant (*p* ≤ *0.05*) restoration of SOD, GPx and GR activities with 1.44, 1.73 and 1.29 fold increases, respectively, as compared with NDEA group. Further, GSH levels were also significantly (*p* ≤ *0.05*) increased in NDEA + BIX group as compared with NDEA group. The BIX alone‐treated group showed no significant changes in antioxidant enzymatic and non‐enzymatic activities as compared with CONTROL group (Table 3).

**Table 3:** Effect of BIX on antioxidant defense status.

Parameter	CONTROL	BIX	NDEA	NDEA + BIX
Superoxide dismutase (SOD) (IU/min/mg protein)	0.16 ± 0.02	0.16 ± 0.02	0.09 ± 0.03^a^, ^b^	0.13 ± 0.01^c^
Glutathione peroxidase (GPx) (nmol/min/mg protein)	2.01 ± 0.03	1.97 ± 0.02	0.90 ± 0.06^a^, ^b^	1.56 ± 0.09^a^, ^b^, ^c^
Glutathione reductase (GR) (nmol/min/mg protein)	1.56 ± 0.12	1.62 ± 0.06	0.71 ± 0.01^a^, ^b^	1.03 ± 0.01^a^, ^b^, ^c^
Reduced Glutathione (GSH) (nmol/min/mg protein)	3.77 ± 0.06	3.72 ± 0.12	1.72 ± 0.16^a^, ^b^	3.02 ± 0.21^a^, ^b^, ^c^

*Note:* Data were expressed as mean ± S.D and analyzed using one‐way ANOVA followed by Tukey's HSD post hoc test. Symbols represents statistical significance (*p* ≤ *0.05)*:

^a^ w.r.t. CONTROL;

^b^ w.r.t. BIX group;

^c^ w.r.t. NDEA group.

## 4 Discussion

BIX is an ER stress modulator that selectively induces the expression of GRP78/BiP, a central ER‐resident chaperone responsible for maintaining protein homeostasis [20]. It has been reported that BIX enhances the ER's capacity to handle misfolded proteins, thereby preserving ER function [35]. The ER modulating potential of BIX was well demonstrated in various pathological conditions like diabetes, stroke, retinal neurodegeneration, and osteoporosis, thereby restoring ER activity [17, 24, 36, 37]. In the present study, we evaluated the protective effect of BIX during the initiation stage of hepatocarcinogenesis.

To experimentally model the initiation stage of hepatocarcinogenesis, a single dose of a well‐established hepatocarcinogenic agent, NDEA, was administered to Wistar rats. NDEA undergoes metabolic bioactivation by cytochrome P450 enzymes located in the endoplasmic reticulum, resulting in the generation of highly reactive metabolites such as ethyl diazonium ion or ethylcarbonium [38]. These electrophilic alkylating agents attack nucleophilic centers in DNA strands, leading to the formation of alkylated or ethylated molecular adducts, which are key events of the initiation of carcinogenesis [39]. The formation of alkylated DNA can destabilize the DNA structure, leading to various lesions, including single‐strand breaks (SSBs) [40]. This structural disruption also makes DNA bases more susceptible to oxidative damage, resulting in the formation of 8‐hydroxy‐2′‐deoxyguanosine (8‐OHdG). Therefore, elevated 8‐OHdG levels serve as a key marker of DNA damage from carcinogen assault [41]. Previous studies in NDEA‐induced hepatocarcinogenesis models have demonstrated that hepatic 8‐OHdG levels remain maintained during the early 24–72 h period following NDEA administration and that cumulative oxidative DNA damage within this interval correlates with subsequent development of preneoplastic hepatic foci, supporting its relevance to the initiation phase of hepatocarcinogenesis [42, 43, 44, 45, 46, 47]. Further, a single dose of NDEA has been shown to induce early genotoxic and pro‐tumorigenic changes within this time frame, which later progress towards hepatocellular carcinoma (HCC) [4, 48]. Therefore, the 48 h time point was selected to evaluate sustained early genotoxic and initiation‐associated molecular alterations rather than only immediate acute toxic injury. Consistent with this, our findings showed significantly increased levels of 8‐OHdG in NDEA group as compared to the CONTROL group, indicating extensive DNA damage after NDEA exposure. Further, these genotoxic lesions activate tumor suppressor genes like p53, which regulate DNA repair, cell cycle arrest, and apoptosis to maintain genomic stability [49]. However, if the repair mechanism fails, persistent lesions can lead to permanent mutations in GC base pairs of oncogenes and tumor suppressor genes [50]. These unrepaired DNA lesions subsequently activate the DNA damage response through kinases such as ATM (ataxia telangiectasia mutated) and ATR (ataxia telangiectasia and Rad3‐related), which transmit damage signals from the nucleus to cytoplasmic signaling complexes involved in inflammatory regulation [51]. One important downstream consequence is the activation of the IκB kinase (IKK) complex. The IKK complex mediates the phosphorylation of IκBα, a regulatory protein that maintains NF‐κB in an inactive state within the cytoplasm. Phosphorylation‐dependent degradation of IκBα facilitates the nuclear translocation of NF‐κB, leading to increased transcription of inflammatory mediators, including TNF‐α and IL‐6 [52]. This sustained cytokine signaling further activates downstream pathways such as STAT3 and MAPK, thereby promoting cell survival and proliferation [53]. Consistent with this, in the present study, NDEA administration demonstrated significantly increased p53, TNF‐α, IL‐6, and PCNA levels in the NDEA‐treated group in comparison with the CONTROL group, indicating enhanced p53 expression, inflammation, and cellular proliferation. Collectively, these coordinated events indicated that early molecular alterations in hepatic tissue were associated with NDEA exposure.

Surprisingly, BIX administration to the animals inhibited these early molecular alterations associated with NDEA exposure. This effect might be attributed to the inhibition of ER stress by BIX. Previous studies demonstrated that BIX induces *GRP78/BiP* expression predominantly through activation of the ATF6 pathway, where BiP mRNA induction becomes evident as early as 6 h after administration and remains elevated for up to 24 h [20]. Further, several studies have also reported that exposure to ER stress‐modulating agents over a period of a few days to weeks might be necessary to effectively alleviate ER stress and its associated pathological consequences [21, 24, 54]. Therefore, here in this present study, the repeated administration of BIX during the 2‐week pre‐treatment period served as a pre‐conditioning phase, wherein the elevated GRP78/BiP levels enhanced the ER's capacity to mitigate severe proteotoxic stress induced by NDEA exposure. This, in turn, limits excessive activation of UPR, thereby attenuating downstream inflammatory, oxidative, and genotoxic responses that contribute to cellular injury and tumorigenic progression.

ER stress significantly influences the DNA repair mechanisms through activation of the UPR [55, 56]. Especially, the activation of the PERK‐eIF2α signaling pathway suppresses global protein translation as part of an adaptive stress response, which reduces the synthesis of essential DNA repair proteins [57]. Consequently, impaired production of DNA repair factors compromised the cellular ability to efficiently repair damaged DNA, thereby promoting genomic instability.

Further, BIX also inhibited UPR‐driven pro‐tumorigenic stress signaling activated by NDEA‐induced UPR. This was well reflected in the NDEA + BIX‐treated group, where the expression of UPR markers (*PERK, ATF6, CHOP*, and the p‐PERK/PERK ratio) was significantly decreased as compared to the NDEA group, highlighting the potential of BIX to attenuate UPR activation. Notably, although BIX is known to enhance *GRP78/BiP* expression *via* activation of the ATF6 pathway, previous studies have demonstrated that BIX induces a transient and adaptive activation of the ATF6‐BiP/GRP78 axis rather than prolonged UPR activation [20]. Therefore, the decreased expression of *ATF‐6* in the NDEA + BIX group might indicate an overall reduction in ER stress burden, consistent with attenuation of UPR signaling and restoration of ER homeostasis, rather than direct suppression of ATF6 signaling.

Several reports have clearly demonstrated that activation of the UPR during unresolved ER stress triggers multiple downstream signaling pathways, including NF‐κB‐mediated inflammatory responses, JNK/MAPK stress signaling, PI3K‐AKT survival pathways, and STAT3 signaling that might drive malignant transformation [58, 59, 60, 61]. However, previous studies have shown that during acute liver injury, activation of these pathways primarily reflects adaptive inflammatory and regenerative responses aimed at restoring hepatic homeostasis rather than direct oncogenic signaling [48, 62, 63]. Nevertheless, these reparative responses might create a tumor‐permissive microenvironment, allowing hepatocytes harboring genetic alterations to survive and clonally expand, thereby contributing to the early molecular events underlying hepatocarcinogenesis [64]. Interestingly, BIX treatment significantly decreased the expression of pro‐inflammatory cytokines (IL‐6 & TNF‐α) and increased the expression of anti‐inflammatory cytokines as compared to the NDEA group. These findings suggested that attenuation of UPR activation by BIX limited the downstream activation of inflammatory signaling pathways associated with ER stress, thereby reducing the inflammatory milieu associated with early hepatocarcinogenic events. Previous studies have also identified IL‐6 as a key mediator of compensatory hepatocyte proliferation following toxic liver injury [62, 65]. This was well reflected in the present study, where attenuation of the inflammatory response by BIX was accompanied by a significant decrease in PCNA expression in the NDEA + BIX group as compared to NDEA group, indicating a reduced requirement for activation of regenerative proliferative responses and a diminished opportunity for expansion of initiated hepatocytes. Furthermore, p53 expression was also significantly decreased in the BIX‐treated group as compared to the NDEA group, indicating that attenuation of NDEA‐induced ER stress and cellular injury might reduce the requirement for activation of p53‐mediated DNA damage response pathways.

Overall, the findings from the present study demonstrated that modulation of ER activity by BIX confers significant protection during the initiation stage of NDEA‐induced hepatocarcinogenesis. This was clearly demonstrated in our findings, where BIX treatment effectively inhibited UPR activation, thereby attenuating early molecular alterations. This consequently preserved the synthesis of essential DNA repair factors, thereby maintaining cellular capacity to efficiently repair damaged DNA and prevent genomic instability. Further, BIX treatment significantly decreased the expression of pro‐inflammatory cytokines, reduced abnormal cellular proliferation, and restored the expression of tumor suppressor protein p53. These findings were further supported by histopathological observations, which demonstrated improved hepatic architecture in the BIX‐treated group as compared with the NDEA group. Additionally, administration of BIX alone did not produce significant alterations in liver injury markers, antioxidant status, inflammatory mediators, or hepatic histoarchitecture when compared with the CONTROL group. These findings suggested that BIX, at the dose employed in the present study (0.1 mg/kg b.w), does not exert detectable basal hepatotoxic effects. The absence of adverse biochemical or histological alterations in the BIX‐alone group further supported the potential translational relevance of BIX as a safe ER‐modulating chemopreventive agent. Collectively, these findings suggest that enhancement of ER adaptive capacity by BIX attenuates key pathways associated with early hepatocarcinogenic progression.

Although the present study provides insights into the effect of modulating ER activity during the initiation stage of hepatocarcinogenesis, the long‐term effect on tumor progression and malignant transformation remains to be elucidated. Therefore, future studies investigating targeted ER modulation during the advanced stage of tumor development might reveal whether maintaining ER homeostasis could effectively inhibit hepatocarcinogenesis, limit tumor growth, and thereby provide a mechanistic rationale for developing ER‐targeted strategies as a potential approach for preventing hepatocellular carcinoma.

## Author Contributions

**Maya P. Shetty:** investigation, methodology, writing – original draft, software, data curation, formal analysis. **Smita Hegde:** supervision, methodology. **Sanjay Bharati:** conceptualization, funding acquisition, writing – review and editing, project administration, supervision, resources, formal analysis, visualization.

## Ethics Statement

All animals were handled according to Committee for the Purpose of Control and Supervision of Experiments on Animals (CPCSEA) guidelines, Government of India. Institutional animal ethics committee approval no IAEC/KMC/119/2022.

## Conflicts of Interest

The authors declare no conflicts of interest.

## Data Availability

The data that support the findings of this study are available from the corresponding author upon reasonable request.
